# Exploring the association between erythema multiforme and HIV infection: some mechanisms and implications

**DOI:** 10.1186/s12981-024-00607-6

**Published:** 2024-04-18

**Authors:** Shumani Charlotte Manenzhe, Razia Abdool Gafaar Khammissa, Sindisiwe Londiwe Shangase, Mia Michaela Beetge

**Affiliations:** 1 Department of Periodontics and Oral Medicine, School of Dentistry, University of Pretoria, PO Box 1266, Pretoria, 0001 South Africa; 2 School of Dentistry, University of Pretoria, Pretoria, South Africa

**Keywords:** HIV, Erythema Multiforme, Adverse drug reactions, HAART, Polypharmacy, Mucocutaneous lesions

## Abstract

Erythema multiforme (EM) is an immune-mediated mucocutaneous condition characterized by hypersensitivity reactions to antigenic stimuli from infectious agents and certain drugs. The most commonly implicated infectious agents associated with EM include herpes simplex virus (HSV) and *Mycoplasma pneumoniae*. Other infectious diseases reported to trigger EM include human immunodeficiency virus (HIV) infection and several opportunistic infections. However, studies focusing on EM and human immunodeficiency virus (HIV) infection are scarce. even though the incidence of EM among HIV-infected individuals have increased, the direct and indirect mechanisms that predispose HIV-infected individuals to EM are not well understood. In turn, this makes diagnosing and managing EM in HIV-infected individuals an overwhelming task. Individuals with HIV infection are prone to acquiring microorganisms known to trigger EM, such as HSV, Mycobacterium tuberculosis, Treponema pallidum, histoplasmosis, and many other infectious organisms. Although HIV is known to infect CD4 + T cells, it can also directly bind to the epithelial cells of the oral and genital mucosa, leading to a dysregulated response by CD8 + T cells against epithelial cells. HIV infection may also trigger EM directly when CD8 + T cells recognize viral particles on epithelial cells due to the hyperactivation of CD8 + T-cells. The hyperactivation of CD8 + T cells was similar to that observed in drug hypersensitivity reactions. Hence, the relationship between antiretroviral drugs and EM has been well established. This includes the administration of other drugs to HIV-infected individuals to manage opportunistic infections. Thus, multiple triggers may be present simultaneously in HIV-infected individuals. This article highlights the potential direct and indirect role that HIV infection may play in the development of EM and the clinical dilemma that arises in the management of HIV-infected patients with this condition. These patients may require additional medications to manage opportunistic infections, many of which can also trigger hypersensitivity reactions leading to EM.

## Introduction

Erythema multiforme (EM) is an acute immune-mediated mucocutaneous blistering disease that may affect up to 1% of the population at some time of their life, particularly in subjects between 20 and 40 years of age, and males more frequently than females. Typically, skin lesions manifest as erythematous ‘target’ or ‘iris’ lesions in which the ‘bulls eye’ of the target quite rapidly becomes a vesicle/bulla. Lesions are usually distributed bilaterally symmetrically on the extremities and face [1, 2]. The oral mucosa is involved in up to 70% of subjects with EM and may be the only affected site. Oral EM can affect any part of the non-keratinized oral mucosa [3], with a predilection for the anterior part of the mouth [4], where it appears as erythematous macules that rapidly become blisters and rupture, giving rise to diffuse multifocal erosions or superficial ulcers on erythematous bases [2]. The lips are invariably hyperemic, eroded, or ulcerated, and swollen lips split, bleed, and become crusted. Oral EM is painful, interfering with eating, swallowing and speech [2, 3].

Most cases of EM are associated with viral infections, particularly with herpes simplex virus (HSV) and human immunodeficiency virus (HIV) infections [5], but also with *Mycoplasma pneumonia* or with drugs such as nonsteroidal anti-inflammatory agents, sulfonamides, barbiturates, food additives such as benzoates, and certain industrial chemicals [4].

The pathogenesis of EM is not well understood. The deposition of immune complexes in the arterioles and capillaries of the lamina propria/dermis and cytotoxic CD8 + T cell immune responses to exogenous antigens such as HSV DNA fragments or reactive drug metabolites within the epithelium play a fundamental role in the initiation and progression of EM [2, 6]. Reactive CD8 + T cells, through the agency of perforin and granzyme, may directly kill or mediate apoptosis of oral and other epithelial cells [3]. Furthermore, damaged epithelial cells may illicit the release of proinflammatory cytokines into their microenvironment, and the consequent inflammatory reaction exaggerates tissue damage [7].

Although apoptosis of epithelial cells may be an event of some importance in the pathogenesis of early or ‘target’ lesion phase of erythema multiforme. The intense inflammatory reaction observed in more advanced lesions suggests that necrosis of keratinocytes is associated with cytotoxic CD8 + T cells and exaggerated expression of local cytokines, rather than apoptosis per se, is the predominant pathogenic event in oral EM [8, 9].

The purpose of this narrative review is to discuss the potential direct and indirect impact HIV infection has on the development of EM; highlighting the importance of interprofessional collaboration in the management of HIV infected individuals who often require additional therapy in the form of drugs known to trigger EM. Information for this article was obtained by employing PubMed and MEDLINE database search engines using the terms HIV, erythema multiforme, adverse drug reactions, HAART, polypharmacy, and mucocutaneous lesions and by analyzing references from relevant widely published articles that were deemed pertinent. The objective of this review was to scrutinize academic papers written in English. No limitations were imposed regarding the location, time period of the papers under examination or number of papers scrutinised.

### The role of HIV in the development of erythema multiforme

The frequency of EM in HIV-positive patients has increased and these patients present with atypical symptoms and persistent skin lesions. Most cases of EM are associated with viral infections, particularly herpes simplex virus (HSV), Epstein-Barr virus (EBV), and HIV [4, 5]. HIV infection plays direct and indirect roles in triggering EM. The indirect role of HIV infection in the development of EM can be attributed to the immune system’s response to HIV infection, synergistic interactions with other infections (such as *Mycoplasma pneumoniae*, Mycobacterium tuberculosis (TB), Treponema pallidum, and histoplasmosis), drugs (such as nonsteroidal anti-inflammatory agents, sulfonamides, and barbiturates), food additives (such as benzoates), and certain industrial chemicals [6, 10, 11]. The possible reason for this may be due to the dysregulation of CD8 + T cells seen in HIV infection and the direct interaction between HIV and epithelial cells.

#### Hyperactivation of CD8 + T cells

HIV infection results in a dysfunctional immune response with disrupted T cell homeostasis, marked by a decreased CD4+:CD8 + T cell ratio [3]. Progression of HIV infection is characterized by a gradual decline in CD4 + T cells and rapid expansion and activation of CD8 + T-cells at the onset and during the chronic phase of HIV infection [3]. The low CD4+:CD8 + T-cell ratio increases the risk of hypersensitivity drug reactions due to the hyperactivation of CD8 + T cells [3, 12–15]. Interestingly, EM has been reported as a sign and symptom of acute HIV infection as part of a seroconversion illness [16–18], a time when there is rapid expansion and activation of CD8 + T cells. The expansion and activation of CD8 + T-cells is also observed after HIV rebounds, with an increase in viral load following a period of viral suppression, due to factors including interruption of ART [13] or reinfection. The interruption of ART is expected to result in an increase in viral load and a low CD4+:CD8 + T-cell ratio. Discontinuation of ART and a low CD4+: CD8 + T-cell ratio have been associated with EM due to the expansion and activation of CD8 + T cells [1, 19].

The declining CD4 + T cells have a significant influence on the way HIV infection is managed, with a focus on decreasing the viral load and reconstituting CD4 + T cells [3, 14, 20]. The latter is used as a surrogate marker for immune reconstitution, with little or no attention given to the consequence of increased dysfunctional and dysregulated CD8 + T cells [3, 14, 15]. This is despite the fact that hyperactivation of CD8 + T cells is considered a hallmark of chronic HIV infection and HIV rebound [13]. The activation of these CD8 + T cells involves nonspecific mechanisms, which include cross-reactivity and antigen-independent cytokine activation, given the propensity of these cells to stimulate cytokines [13]. The T cells involved are mainly non-HIV-specific CD8 + T cells because of the so-called bystander activation [13, 21].

The persistent increase and activation of CD8 + T cells is reported to correlate proportionally with an increased risk of non-AIDS-related morbidity and mortality, which is linked to inflammation despite reconstitution of CD4 + T cells with antiretroviral therapy [14, 20]. The non-AIDS related conditions are linked to activation of non-HIV specific CD8 + T cells directed against non-persistent and persistent antigens derived from new or latent viral and bacterial infections [13, 14, 20]. Viruses inducing CD8 + T cell activation include HSV, CMV, EBV, Influenza virus, and adenovirus, leading to non-AIDS-related events [3, 13, 21]. These viruses are common in HIV-infected individuals. This corroborates the increased risk of EM among HIV-infected individuals, since the viruses mentioned are also triggers of EM [22, 23]. Bacteria and their by-products can also promote non-HIV-specific CD8 + T cell activation when translocated to an injured site [20]. The bacterial products found to be associated with hyperactivation include lipopolysaccharides (LPS) [13], which are important antigens for periodontal pathogens. Thus, anaerobic bacteria producing LPS found in dental biofilms could be a potential culprit in the development of EM. Poor oral hygiene can predispose HIV-infected individuals to EM.

The hyperactivation of CD8 + T cells may explain the link between HIV infection and EM. Exposure to HIV and ART in susceptible individuals triggers a dysregulated CD8 + T cell-mediated immune reaction in keratinocytes. Dysregulated CD8 + T cells against persistent and non-persistent antigens are fertile grounds for EM development. This is further supported by the fact that in the subset of non-HIV-specific CD8 + T cells, memory cells are activated more efficiently than naïve cells [24], indicating a more robust response leading to the destruction of keratinocytes seen in EM. There is also potential cross-reactivity of non-HIV CD8 + T cells directed against other infectious agents for HIV virions on infected keratinocytes or that of HIV-specific CD8 + T cells for infectious antigens other than HIV infection [24]. The potential role of HIV infection in triggering EM could be seen as both a direct and indirect mechanism, given the known immunological response, which involves hyperactivation of CD8 + T cells [13].

Although data on the prevalence of EM among HIV-infected individuals are scarce, it can be presumed that individuals who are HIV-positive and on ART have an increased risk for EM. Hence, EM and its more severe counterparts, Steven Johnson syndrome (SJS) and toxic epidermal necrolysis (TEN), are increasingly being observed among HIV-positive individuals [19]. Underreporting and misdiagnosis of EM among HIV-infected individuals may be a significant contributing factor to the lack of data on the prevalence of EM among HIV-infected individuals. Moreover, the fact that the condition is acute and self-limiting, resolving within weeks without any significant sequelae, means that it can go unnoticed and unreported [10, 16]. The absence of a universally accepted distinction between EM and conditions such as SJS adds to the underreporting of EM incidence in HIV-infected individuals [25].

#### Direct tissue damage: HIV-epithelial cell interaction

HIV-epithelial cell interactions significantly influence the pathogenesis of HIV-related diseases. HIV can interact with epithelial cells in the genital and oral mucosa during both its initial encounter and the spread of systemic HIV infection [26]. Independent of CD4, HIV is known to infect and bind to epithelial cells through alternative HIV-associated receptors, including C-X chemokine receptor type 4 (CXCR4), C-C chemokine receptor type 5 (CCR5), galactosylceramide (GalCer), heparan sulfate proteoglycans (HSPG), mannose receptors, and T cell immunoglobulin and mucin domain 1 (TIM-1) [7, 26]. When HIV comes into contact with epithelial cells, the integrity of the barrier is compromised, leading to the internalization of the virus [26].

We postulate that comparable mechanisms could amplify EM in the context of both local and systemic immunoinflammatory reactions targeting HIV-infected epithelial cells. Furthermore, HIV interaction with epithelial cells results in an increased expression of the cytokines tumour necrosis factor alpha (TNF-α) and interferon gamma (IFN-γ) [26], which is believed to play a role in the pathogenesis of EM.

#### Dysfunctional Regulatory T-cells (Tregs)

An additional class of T cells, regulatory T cells (Tregs), have a protective function against the development of adverse drug reactions on the skin. Previous research has shown that functional CD4 + CD25 + Tregs help reduce inflammation and maintain an immune balance by regulating the magnitude of immune responses by suppressing both CD4 + and CD8 + T cell activation and function. Dysfunctional Tregs, however, are associated with adverse drug reactions and the development of immune-mediated conditions [28], such as EM. This is because Tregs, play a crucial role in mediating immunologic self-tolerance. A decrease in CD4 + T cells in HIV-positive individuals has been linked to an increased risk of inflammatory skin conditions owing to the depletion of CD4 + CD25 + Tregs [15].

#### Presence of opportunistic infections

Individuals with HIV are more likely to develop opportunistic infections owing to a gradual decline in CD4 + T cells. These include infectious agents that are also implicated in EM, including HSV, Epstein-Barr virus (EBV), varicella zoster virus (VZV), hepatitis C virus, cytomegalovirus (CMV), streptococcal, Mycobacterial tuberculosis, Treponema pallidum, and histoplasmosis [10, 11, 19, 27–29]. Despite the significant decline in the incidence of these types of infections since the advent of ART, they continue to occur in a considerable number of HIV-infected patients. The activation of a subset of non-HIV-specific CD8 + T memory cells against epithelial cells is more robust and efficient in the presence of these infectious agents than in their naïve counterparts [24]. HIV and these infectious agents have a synergistic interaction within the epithelia, which can accelerate the development of EM. This interaction involves disruption of the epithelial barrier caused by HIV’s interaction of HIV with epithelial cells, which facilitates the acquisition and/or activation of opportunistic infections. This leads to an inflammatory response that induces the release of HIV virions from infected epithelial cells and a resulting CD8 + T cell response that is implicated in the development of EM. This vicious cycle created by this synergistic interaction could be one of the reasons for the increased incidence and persistence of EM among HIV-infected individuals.

#### Adverse reaction to antiretrovirals

Adverse drug reactions (ARDs) are defined as harmful and unintended responses to medication at typical therapeutic doses. These reactions can manifest as side effects, allergic reactions, or other adverse events [30]. Erythema multiforme (EM) is a short-term adverse effect that may go unnoticed in HIV-infected individuals but can become more pronounced when ART is introduced [9, 31]. EM is reported to be a common cutaneous adverse drug reaction in HIV-infected individuals, with drugs triggering approximately 50% of cases [32]. In HIV-infected patients, the prevalence of EM is even higher and has a more severe clinical presentation [9]. The use of ART for the treatment of HIV infection is associated with a range of ARDs, from mild discomfort to severe life-threatening side effects [9, 33, 34]. Antiretroviral drugs known to cause EM include zidovudine, abacavir, efavirenz, nevirapine, protease inhibitors, etravirine, tenofovir, and new antiretrovirals [8, 9, 34, 35]. Concurrent use of these drugs with other medications that can trigger EM, such as penicillin’s, cephalosporins, macrolides, sulphonamides, antipyretics, cotrimoxazole, isoniazid, nonsteroidal anti-inflammatory drugs (NSAIDs), and herbal remedies, can exacerbate the condition [1, 4, 9, 22, 36].

The risk of hypersensitivity drug reactions increases when the CD4+:CD8 + T-cell ratio is low, leading to the hyperactivation of CD8 + T-cells, which is important in delayed hypersensitivity reactions involved in the pathogenesis of EM [10, 16, 24]. The reaction towards certain ART metabolites triggers a dysregulated T cell response against epithelial cells expressing antigens from infectious agents and drug haptens, resulting in an influx of CD8 + T cells, macrophages, and neutrophils that release a range of cytokines [9, 36]. These cytokines, along with those released by targeted epithelial cells, mediate inflammation [4, 9, 10]. The resulting inflammation leads to epithelial cell death, sometimes accompanied by sub- and intra-epithelial vesiculation, and ultimately results in the blistering, erosion, and ulceration seen in EM [4, 8, 37].

Considering the complex array of medications that most HIV-positive patients typically take, it can be challenging to accurately confirm adverse drug reactions (ADR). One such ADR is a reaction with eosinophilia and systemic symptoms (DRESS), a delayed-type hypersensitivity reaction that presents with symptoms such as pruritic maculopapular rash, eosinophilia, lymphadenopathy, and potentially life-threatening conditions like hepatitis, nephritis, and pneumonitis [34, 38]. DRESS is a form of drug-induced hypersensitivity reaction developing within one– six weeks of exposure to the offending drug [34]. In some cases, EM may be mistaken for DRESS because of the similar symptoms. Biopsies are often not performed in these patients, and the diagnosis is typically based on clinical and serological findings. It is possible that some cases initially believed to be DRESS may actually have EM with DRESS-like features. Therefore, EM should be considered in the differential diagnosis of DRESS in HIV-positive patients.

### Diagnosing erythema multiforme in HIV-Positive individuals

Diagnosing EM and identifying potential triggers in HIV-positive individuals can be challenging, given the many possible triggers, including HIV infection, ART, drugs used to treat opportunistic infections or as prophylaxis, and persistent or non-persistent antigens from various infectious agents. A suggested method for identifying drug triggers is the provocation test, in which certain drugs are temporarily stopped and reintroduced to observe any hypersensitivity reactions [9].

EM diagnosis typically relies on clinical presentation, and biopsy is not always necessary. However, histopathology may be helpful in ruling out other diseases and determining the causative factor(s) [3, 4, 25, 37]. Prompt treatment depends on identifying potential inciting agent(s) and eliminating them [3, 25].

While a biopsy can be useful in excluding other conditions with similar clinical presentations [25, 28], histopathology can also reveal the predominance of CD8 + T cells and macrophage infiltrates in EM. Histopathological patterns can be divided into predominantly inflammatory or necrotic patterns depending on the inciting agent [20]. In cases of viral-associated EM, there is a predominantly inflammatory pattern, whereas in drug-associated EM, the predominant pattern is epithelial cell necrosis. Further differentiation between the two categories can be made based on the dominant inflammatory cytokines. Viral-associated EM lesions test positive for TNF-α, while drug-associated EM lesions test positive for IFN-γ, indicating a response to an intracellular antigen [21, 25, 28]. CD8 + T-cells are capable of producing both TNF-α and IFN-γ inflammatory cytokines [26].

### Management of HIV-infected patients with erythema multiforme

The management of EM depends on the severity of the condition, the course of the illness, and the identification of the potential trigger [22, 23, 39]. The management of EM in HIV-positive individuals is similar to that in HIV-negative individuals; however, identifying the potential trigger is crucial. HIV infection, ART, and various infectious agents are potential triggers for EM in HIV-positive individuals, making it difficult to identify the specific drug that may have caused this hypersensitivity reaction. A provocation test in which certain drugs are stopped and then reintroduced to determine the drug triggering the reaction has been suggested [9]. Once the drug has been identified, it should be discontinued, and the patient should avoid re-exposure where possible to prevent cross-reactivity [22, 23].

For acute drug-induced EM, systemic steroids can be used, with patients generally responding well to a short course of systemic corticosteroids at a dose of 40–60 mg/day tapered over 2–4 weeks, depending on the severity [25]. This can be followed by a low maintenance dose or the use of topical corticosteroids. Patients must be carefully monitored for corticosteroid-induced side effects or drug interactions with ART. Some antiretroviral regimens may alter the pharmacokinetics of prednisolone and prednisone; therefore, clinicians should carefully consider these potential interactions and administer the drugs in the safest and most effective manner possible [40].

In acute and recurrent EM associated with HSV, systemic corticosteroids and acyclovir have proven to be effective treatments [22]. For acute EM, acyclovir 200 mg five times daily for five days has been recommended [39]. For recurrent EM, 400 mg acyclovir, 500 mg valacyclovir, or 250 mg famciclovir twice daily for 6 months has been suggested [2, 32]. Topical corticosteroids are suitable in mild cases [23]. For severe recurrent EM resistant to prophylactic antiviral therapy, azathioprine, dapsone, and mycophenolate mofetil may be administered [22, 29].

Interprofessional collaboration is essential to prevent acute drug reactions associated with EM, which can negatively affect patients’ quality of life. Given the potential for polypharmacy and drug interactions in HIV-infected individuals managed in different disciplines, it is crucial to provide supportive care such as a liquid diet, electrolyte supplementation, and, in severe cases, intravenous fluids [3]. Consultation with dieticians or nutritionists may be necessary to assist with balanced meal plans based on the patients’ affordability. For ocular involvement, ophthalmology consultation is necessary for thorough evaluation and treatment to prevent long-term complications [25]. Interprofessional teamwork is crucial to reduce the risk of EM in HIV-infected individuals and ensure holistic management of these patients.

## Conclusion

Underreporting and misdiagnosis of EM among HIV-infected individuals may be a significant contributing factor to the lack of data on the prevalence of EM among HIV-infected individuals. Moreover, the fact that the condition is acute and self-limiting, resolving within weeks without any significant sequelae means it can go unnoticed and not reported. Moreover, the absence of a universally accepted distinction between EM and conditions like SJS and DRESS adds to the underreporting of the incidence of EM in HIV-infected individuals. Although the exact pathogenic mechanism of EM remains unclear, it is widely considered to be a T cell-mediated hypersensitivity reaction with a critical role played by CD8 + T cells. The direct role that HIV plays in precipitating EM require further exploration.

The article highlights the potential direct and indirect role that HIV infection may play in the development of EM and the clinical dilemma that arises in the management of HIV-infected patients with this condition. These patients may require additional medications to manage opportunistic infections, many of which can also trigger hypersensitivity reactions leading to EM. This underscores the limited understanding of EM and its potential triggers, as well as the lack of interprofessional teamwork when managing HIV-infected patients. It is crucial that clinicians consider a patient’s medical history and any other medications they may be taking before prescribing any drug and seek input from colleagues in other relevant disciplines to ensure timely diagnosis and management. An interprofessional team approach is essential for optimal treatment outcomes and improved quality of life in HIV-infected individuals, including the prompt identification of EM cases by all clinicians involved in their care. Early identification of potential triggers for EM can help prevent their recurrence, particularly in cases of polypharmacy.

The declining CD4 + T cells has been a significant influence in the way HIV infection is managed with a focus on decreasing the viral load and reconstituting CD4 + T cells. The latter is used as a surrogate marker for immune reconstitution [28] with little or no attention given to the consequence of increased dysfunctional, and dysregulated CD8 + T cells implicated in several non-AIDS /HIV related conditions. HIV infection should be considered in patients presenting with EM and monitoring the CD4 + T cell:CD8 + T cell ratio should be included in HIV infection control. Further insights into the relationship between EM and HIV could be gained through in-depth observational studies, which could inform a management protocol for HIV-infected patients by a dedicated team.

## Data Availability

Not applicable – Review article.
